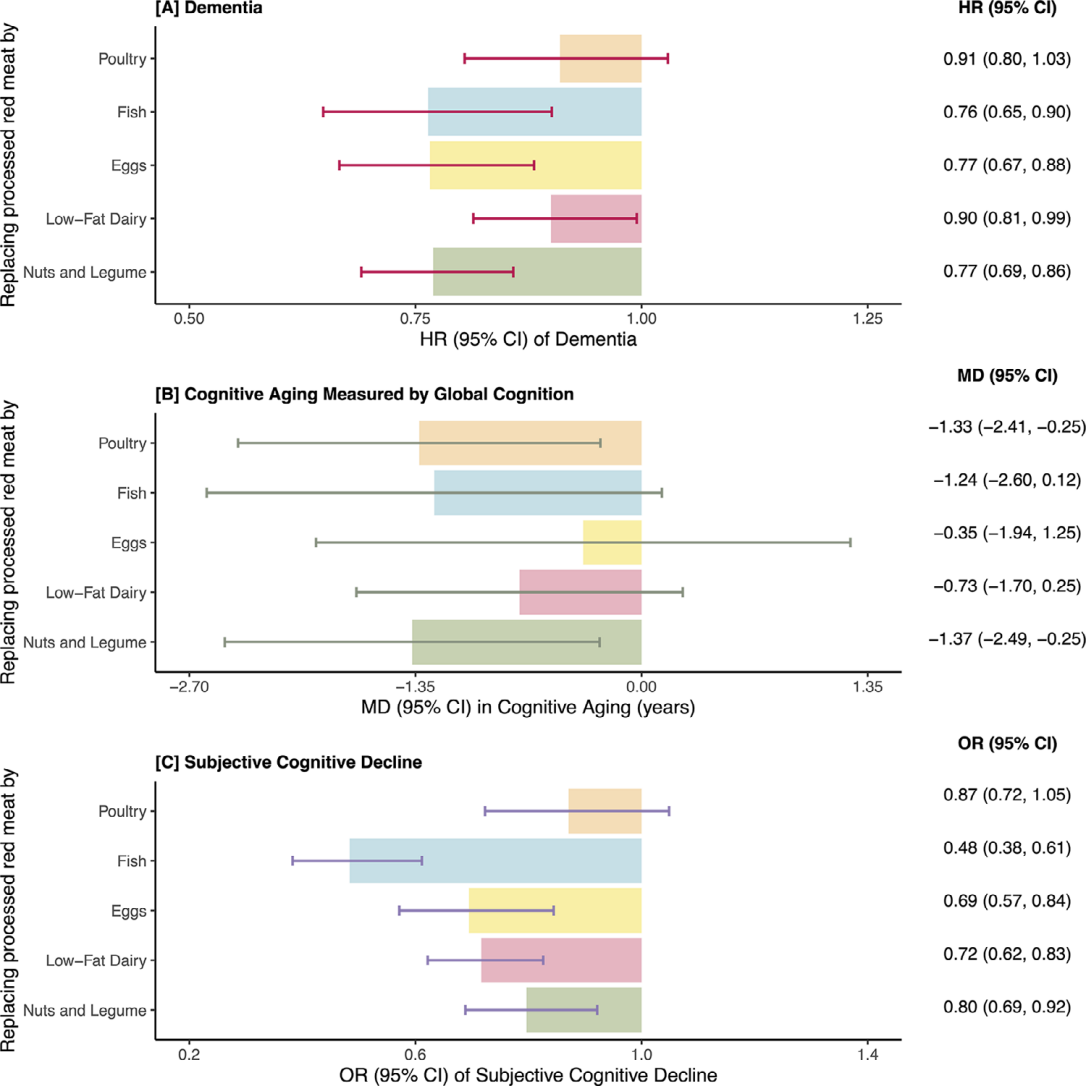# A Prospective Study of Long‐Term Red Meat Intake, Risk of Dementia, and Cognitive Function in US Adults

**DOI:** 10.1002/alz.088556

**Published:** 2025-01-09

**Authors:** Yuhan Li, Yanping Li, Xiao Gu, Yuxi Liu, Danyue Dong, Jae H. Kang, Molin Wang, Heather Eliassen, Walter C. Willett, Meir J. Stampfer, Dong D. Wang

**Affiliations:** ^1^ Harvard T. H. Chan School of Public Health, Boston, MA USA; ^2^ Channing Division of Network Medicine, Brigham and Women’s Hospital and Harvard Medical School, Boston, MA USA; ^3^ Broad Institute of MIT and Harvard, Cambridge, MA USA; ^4^ Channing Division of Network Medicine, Brigham and Women’s Hospital, Harvard Medical School, Boston, MA USA

## Abstract

**Background:**

Previous studies yielded inconsistent results regarding the association between red meat intake and cognitive health. We aimed to prospectively examine the associations between processed and unprocessed red meat intakes and various cognitive outcomes.

**Method:**

We assessed diet intake every 2‐4 years using food‐frequency questionnaires and ascertained incident dementia cases through self‐report and death records in 87,424 participants free from Parkinson’s disease or baseline dementia, stroke, cancer from the Nurses' Health Study (NHS). In a subset of 17,458 NHS participants, cognitive function was assessed using the Telephone Interview for Cognitive Status (1995‐2008). We longitudinally collected information on subjective cognitive decline from 33,908 NHS participants and 10,058 participants in the Health Professionals Follow‐Up Study.

**Result:**

During a follow‐up of 38 years (1980‐2018) in the NHS, we documented 6,856 dementia cases. Participants with processed red meat intake ≥ 0.25 serving/day, as compared to < 0.10 serving/day, had 15% higher risk of dementia (HR = 1.15; 95% CI: 1.08‐1.23; *P*_linearity_ <0.001). We found significant associations between higher processed red meat intake and accelerated aging in global cognition [1.61 years per 1 serving/day increment (95% CI: 0.20, 3.03)] and verbal memory [1.69 years per 1 serving/day increment (95% CI: 0.13, 3.25), both *P*_linearity_ = 0.03]. Participants with processed red meat intake ≥ 0.25 serving/day had a 14% higher likelihood of subjective cognitive decline compared to those with intake < 0.10 serving/day (OR = 1.14; 95% CI: 1.04–1.24; *P*_linearity_ = 0.004), while unprocessed red meat intake of ≥ 1.00 serving/day compared to < 0.50 serving/day was associated with a 16% higher likelihood of subjective cognitive decline (OR = 1.16; 95% CI: 1.04–1.30; *P*_linearity_ = 0.02). In substitution analyses, replacing 1 serving/day of processed red meat with 1 serving/day of nuts and legumes was associated with a 23% lower risk of dementia (HR = 0.77, 95% CI: 0.69–0.86), 1.37 fewer years of cognitive aging (95% CI: ‐2.49—0.25), and a 20% lower odds of subjective cognitive decline (OR = 0.80, 95% CI: 0.69–0.92).

**Conclusion:**

Higher intake of red meat, particularly processed red meat, is associated with a higher risk of developing dementia and worse cognition.